# Fonofos Exposure and Cancer Incidence in the Agricultural Health Study

**DOI:** 10.1289/ehp.9301

**Published:** 2006-08-18

**Authors:** Rajeev Mahajan, Aaron Blair, Charles F. Lynch, Paul Schroeder, Jane A. Hoppin, Dale P. Sandler, Michael C.R. Alavanja

**Affiliations:** 1 Occupational and Environmental Epidemiology Branch, Division of Cancer Epidemiology and Genetics, National Cancer Institute, National Institutes of Health, Department of Health and Human Services, Rockville, Maryland, USA; 2 Department of Epidemiology, University of Iowa, Iowa City, Iowa, USA; 3 Westat, Rockville, Maryland, USA; 4 Epidemiology Branch, National Institute of Environmental Health Sciences, National Institutes of Health, Department of Health and Human Services, Research Triangle Park, North Carolina, USA

**Keywords:** agriculture, fonofos, insecticides, neoplasms, occupational exposure, organophosphorus compounds, organothiophosphorus compounds, pesticides

## Abstract

**Background:**

The Agricultural Health Study (AHS) is a prospective cohort study of licensed pesticide applicators from Iowa and North Carolina enrolled 1993–1997 and followed for incident cancer through 2002. A previous investigation in this cohort linked exposure to the organophosphate fonofos with incident prostate cancer in subjects with family history of prostate cancer.

**Objectives:**

This finding along with findings of associations between organophosphate pesticides and cancer more broadly led to this study of fonofos and risk of any cancers among 45,372 pesticide applicators enrolled in the AHS.

**Methods:**

Pesticide exposure and other data were collected using self-administered questionnaires. Poisson regression was used to calculate rate ratios (RRs) and 95% confidence intervals (CIs) while controlling for potential confounders.

**Results:**

Relative to the unexposed, leukemia risk was elevated in the highest category of lifetime (RR = 2.24; 95% CI, 0.94–5.34, *p*_trend_ = 0.07) and intensity-weighted exposure-days (RR = 2.67; 95% CI, 1.06–6.70, *p*_trend_ = 0.04), a measure that takes into account factors that modify pesticide exposure. Although prostate cancer risk was unrelated to fonofos use overall, among applicators with a family history of prostate cancer, we observed a significant dose–response trend for lifetime exposure-days (*p*_trend_ = 0.02, RR highest tertile vs. unexposed = 1.77, 95% CI, 1.03–3.05; RR_interaction_ = 1.28, 95% CI, 1.07–1.54). Intensity-weighted results were similar. No associations were observed with other examined cancer sites.

**Conclusions:**

Further study is warranted to confirm findings with respect to leukemia and determine whether genetic susceptibility modifies prostate cancer risk from pesticide exposure.

Fonofos (*O*-ethyl *S*-phenyl ethylphosphonodithioate), first registered for use with the U.S. Environmental Protection Agency (EPA) in 1967, is an organophosphate insecticide applied to soil to protect mainly corn but also sugarcane, peanuts, tobacco, and several other crops from various types of worms (U.S. EPA 1999). Fonofos has no residential uses. In 1998, the registrant of fonofos voluntarily cancelled its registration (U.S. EPA 1999).

The current state of knowledge regarding the health effects of fonofos is limited. The few studies examining the genotoxicity and mutagenicity of fonofos have had inconsistent results. Gentile et al. (1982) found fonofos to be mutagenic both directly and after metabolic activation in assays using both *Saccharomyces cerevisiae* and *Salmonella typhimurium*, but Simmons et al. (1979) found it to be negative in both assays.

To our knowledge, there have been no studies of fonofos in whole animals published in the peer-reviewed literature. However, several proprietary 2-year feeding studies conducted for regulatory evaluation found no tumors in rats and mice from the administration of fonofos (California Department of Pesticide Regulation 1997).

The epidemiologic evidence linking fonofos with cancer is suggestive, but insufficient to establish a causal relationship. Early case–control studies of non-Hodgkin lymphoma (NHL) and leukemia risk factors among farmers pointed toward the class of organophosphates (Brown et al. 1990; Cantor et al. 1992; Clavel et al. 1996; Zahm et al. 1993). Later, to evaluate specific pesticides as risk factors for NHL, Waddell et al. (2001) and De Roos et al. (2003) pooled three population-based case–control studies conducted in the midwestern United States and found odds ratios (ORs) for fonofos exposure of 1.7 [95% confidence interval (CI), 1.1–2.6] and 1.8 (95% CI, 0.9–3.5), respectively. Further, because NHL is associated with a compromised immune system, Lee et al. (2004) pooled two of the studies to evaluate risk from pesticide exposure and asthma, a marker of immune alteration, and observed an elevated main effect for fonofos use (OR = 1.6; 95% CI, 1.0–2.4) as well as an elevated joint effect of fonofos use and asthma (OR = 3.7; 95% CI, 1.3–10.9).

In addition, in a tumor-specific analysis of the Agricultural Health Study (AHS), fonofos has been significantly associated with prostate cancer among applicators with a family history of prostate cancer (OR = 1.80; 95% CI, 1.14–2.84) (Alavanja et al. 2003). Moreover, other chemically similar pesticides of the organothiophosphate subclass have also been associated with prostate cancer and lung cancer in tumor-specific analyses (Alavanja et al. 2003, 2004).

In light of the evidence linking fonofos to prostate cancer in the previous tumor-specific analysis and the literature linking fonofos to leukemia and NHL, our primary aim was to study fonofos use with respect to incident cancer of all types in a chemical specific analysis among 45,372 participants of the AHS. Our secondary aim was to use more detailed exposure information along with an additional 3.2 years of follow-up since the previous prostate cancer analysis, which provided 87 new prostate cancer cases with prostate cancer family history information, to see if the prostate cancer finding held.

## Materials and Methods

### Enrollment and follow-up

The AHS has been described previously (Alavanja et al. 1996). Briefly, it is a prospective cohort of 52,395 private applicators (farmers) from Iowa and North Carolina and 4,916 commercial applicators (employees of pest control companies or businesses whose primary function is not pesticide application) from Iowa licensed to apply restricted use pesticides. This cohort represents 82% of eligible applicators from both states during the enrollment period of the study (13 December 1993 to 31 December 1997). Population-based cancer registries of both states were used to identify subjects with incident cancer diagnoses between enrollment and 31 December 2002. Subjects who died or moved out of the state were censored in the year of occurrence of either event. Vital status was ascertained using state death registries and the National Death Index. Residence information was obtained through motor vehicle records, pesticide registration records, and address files of the Internal Revenue Service. To date, average follow-up time is 7.5 years and follow-up is > 98% complete. All participants provided informed consent, and the research protocol was approved by all appropriate institutional review boards.

### Exposure assessment

On enrollment, pesticide applicators seeking a restricted-use pesticide license completed a self-administered questionnaire. The questionnaire obtained detailed exposure information (days of use per year, years of use, and decade of first use) for 22 pesticides (including fonofos) and ever/never use information for 28 additional pesticides, as well as information on application methods, pesticide mixing status, personal protective equipment use, personal equipment repair, smoking, current alcohol use, cancer history in first-degree relatives, and basic demographic data. For some analyses, we used information on solvent exposure that was collected using a self-administered take-home questionnaire completed by 44% of those enrolled [both questionnaires available at http://www.aghealth.org/questionnaires.html (AHS 2006)].

We estimated fonofos exposure in terms of cumulative fonofos lifetime exposure-days and intensity-weighted exposure-days. We calculated lifetime exposure-days as the cross-product of the questionnaire categories for frequency of fonofos use in an average year and the number of years of fonofos use, using the midpoints of the questionnaire categories. We assessed intensity of exposure using an algorithm developed by Dosemeci et al. (2002). This algorithm calculates an intensity score that takes into account the effect of modifying factors, such as how often an applicator personally mixed or prepared the pesticide, the type of application methods used, whether an applicator personally repaired pesticide application equipment, and the type of personal protective equipment used. By multiplying the intensity score with fonofos lifetime exposure-days, we obtained fonofos intensity-weighted exposure-days.

### Statistical analysis

All pesticide applicators who returned an enrollment questionnaire were eligible for analysis. We excluded applicators if they did not provide information on fonofos exposure duration (*n* = 5,987); if their first primary cancer occurred before enrollment (*n* = 937), if they did not live in either state on enrollment (*n* = 295); or if they did not provide information on birth year (*n* = 2), smoking (*n* = 1,664), or use of correlated pesticides (*n* = 3,054). After exclusions, 45,372 subjects were available for the lifetime exposure-days analysis. The intensity-weighted exposure-days analysis contained four fewer cancer cases and 55 fewer cancer-free subjects because of missing data on intensity metric covariates. Compared with retained subjects, excluded subjects were generally older and more likely to be from North Carolina.

We categorized fonofos lifetime exposure-days and intensity-weighted exposure-days into tertiles based on the distribution of exposure among all cancer cases. Because the tertiles of intensity-weighted exposure-days based on the exposure distribution among all cancer cases resulted in categories inadequate for analyzing leukemia (one case in the lowest exposed group), we created more uniform tertiles for leukemia based on the exposure distribution among leukemia cases. We used two different reference groups—the unexposed and the lowest exposed groups—to analyze all cancer sites for which there were at least 15 exposed cases and 4 cases per lifetime exposure-days category. Specifically, we examined all cancers combined; cancers of the prostate, lung, and colon; melanoma skin cancer; leukemia; and lymphohematopoietic cancer consisting of Hodgkin lymphoma, NHL, leukemia, and multiple myeloma.

Statistical analyses were conducted in AHS data release 0412.01 using Stata version 8 (StataCorp 2003). We used Poisson regression to calculate rate ratios (RRs) and 95% CIs while adjusting for multiple covariates. Covariates included category of age at enrollment (< 40, 40–49, 50–59, ≥ 60 years of age), state of residence, pack-years of smoking categorized at the median (0, ≤ 12, > 12), and use of the four most correlated pesticides [trichlorofon, carbofuran, imazethapyr, and *S*-ethyl dipropylthiocarbamate (EPTC)]. Pearson correlation coefficients ranged between 0.43 and 0.50. Use of each correlated pesticide was classified as never, low, and high, with the median lifetime exposure-days categorizing low and high usage. As an alternative strategy to account for use of other pesticides, we also replaced use of the most correlated pesticides with lifetime exposure-days to all pesticides. Further adjustment for education, sex, alcohol consumption in the previous 12 months, applicator type, cancer history in first-degree relatives, and enrollment year did not affect point estimates by > 10%. We performed linear trend tests to assess the overall dose–response trend by entering exposure categories ordinally in the models after assigning them median exposure value in that category. All statistical tests were two-sided.

## Results

Selected characteristics of the study population are displayed in Table 1 according to lifetime exposure-days category. In this table, “lowest exposed” refers to those in the lowest exposure tertile (> 0–20 lifetime exposure-days), whereas “other exposed” refers to those in the middle and highest tertiles (> 20 lifetime exposure-days). Overall, two-thirds of the study participants were from Iowa. More than two-thirds of the cohort reported corn farming. Study subjects were also predominantly white and male. Just over half reported being never smokers. Close to 55% reported that the highest level of schooling attained was no more than a high school diploma. Approximately 40% reported a history of cancer in first-degree relatives.

The unexposed group was generally younger, less likely to use alcohol, and slightly less likely to report a family history of cancer, used fewer pesticides in general, and planted fewer acres than either exposed group. Both Iowa participants and corn farmers were over-represented in the exposed categories. Based on these differences between the unexposed group and either exposed group, the lowest exposed group may represent the exposed group more closely.

With the intensity-weighted metric, risk estimates for all cancers combined were not different from the null, regardless of the reference group used (Table 2). Colon cancer risk estimates were elevated, but only when using the unexposed as the reference, and the relationship was not monotonic. Leukemia risk estimates were elevated regardless of the reference group used. When the unexposed group was the reference, the RR was 2.67 (95% CI, 1.06–6.70) in the highest exposure category, and the test for linear trend was significant (*p*_trend_ = 0.04). When the lowest exposed group was the reference, the corresponding RR was 2.03 (95% CI, 0.58–7.05). The linear trend test was not significant. Fonofos intensity-weighted exposure-days were not related to the risk of any other examined cancer.

Results were similar using the lifetime exposure-days metric (not shown). For example, using the unexposed group as the reference, leukemia RRs increased monotonically to 2.24 (95% CI, 0.94–5.34) in the highest tertile (*p*_trend_ = 0.07). When the lowest exposed tertile was used as the reference, the risk estimates increased monotonically with increasing exposure category to 2.18 (95% CI, 0.57–8.40) in the highest tertile. The test for linear trend was not significant.

To account for the effect of misclassification due to the inclusion of exposure that occurred too recently to affect cancer risk, we repeated the analyses excluding 39 cancer cases and 1,389 cancer-free subjects who either reported first using fonofos during the 1990s or did not provide this information. The results were similar to those presented here (not shown). The results were also similar after repeating the analyses among Iowa participants only (not shown). Additionally, to control for pesticide use in general, we repeated the analyses adjusting for lifetime exposure-days to all pesticides instead of the most correlated pesticides, and the results did not differ from those presented here (not shown). To evaluate the effect of missing information, we repeated the analyses while allowing subjects with missing information on covariates to influence the outcome by assigning them an unspecified category. Once again, the results were largely the same (not shown). Finally, the results were similar when we separately examined fonofos days of use and years of use after categorizing each into none, low, and high categories for exposure, using the median to distinguish between low and high (not shown).

We further investigated leukemia by separately examining chronic lymphocytic (eight exposed cases), chronic myelogenous (two exposed cases), acute myelogenous (five exposed cases), and all other leukemias (three exposed cases) (not shown). Acute lymphocytic leukemia could not be evaluated (no exposed cases). Although the CIs were wide and the point estimates were not significant, relative to the unexposed, the age-adjusted risk estimates in low- and high-exposure categories were elevated for all examined subtypes. In the high-exposure category, point estimates ranged from 1.75 for acute myelogenous to 3.65 for chronic myelogenous leukemia.

We also adjusted leukemia risk estimates using data on use of gasoline, solvents, and paint, which were collected among private applicators using the take-home questionnaire (not shown). Although the subset of otherwise eligible applicators who provided the aforementioned information was small (eight exposed cases), adjusting for these exposures did not weaken the leukemia risk estimates. Finally, controlling for animal exposures using information on the number of livestock (other than poultry) or whether applicators butchered animals, provided veterinary services to livestock, or worked in swine or poultry containing areas, did not affect risk estimates (not shown).

Table 3 shows prostate cancer risk relative to the unexposed using both metrics and stratified by family history of prostate cancer in first-degree relatives. We generated uniform exposure categories based on the exposure distribution among prostate cancer cases. In the group with no prostate cancer family history, risk was not associated with exposure regardless of the metric. In those with a family history of prostate cancer, the risk estimates increased, and significant linear trends were observed using either metric. Using the lifetime exposure-days metric, we observed a significant dose–response relationship (*p*_trend_ = 0.02), which resulted in a RR of 1.77 (95% CI, 1.03–3.05) in the highest exposure category. The interaction term, defined as the cross-product of family history of cancer and category of lifetime exposure-days (treated as a continuous variable), was significant (RR = 1.28; 95% CI, 1.07–1.54). With the intensity-weighted exposure-days metric, risk in the highest category was 1.83 (95% CI, 1.12–3.00). The test for linear trend was significant (*p*_trend_ = < 0.01), as was the interaction RR of 1.27 (95% CI, 1.07–1.51).

When the analysis in Table 3 was repeated using the lowest exposed group as the reference, the results were similar but less pronounced due to decreased statistical power (not shown). Risk was related to fonofos use only in those with a family history of prostate cancer. Point estimates increased monotonically with lifetime exposure-days to 1.24 (95% CI, 0.61–2.51) in the highest category. The interaction RR was 1.25 (95% CI, 0.83–1.89). Point estimates generally increased with intensity-weighted exposure-days to 1.68 (95% CI, 0.83–3.39) in the highest category. The interaction RR was 1.27 (95% CI, 0.85–1.89). Linear trend tests were not significant using either metric.

When the risk of the other examined cancers (all cancers combined, melanoma, leukemia, lymphohematopoietic cancers, lung cancer, and colon cancer) was similarly stratified, no discrepancies were observed comparing those with and without a family history of the specific cancer (not shown).

When we did further analyses to disentangle the effects of prostate cancer family history and fonofos exposure, we observed that the age-adjusted main effect for ever compared with never fonofos exposure was 0.97 (95% CI, 0.80–1.17), whereas for family history of prostate cancer, it was 1.67 (95% CI, 1.35–2.07). The observed joint effect of the two exposures was 2.63 (95% CI, 1.96–3.53).

## Discussion

In this study we evaluated cumulative lifetime fonofos exposure until enrollment as a risk factor for incident cancer occurring between the end of enrollment through 2002. Almost 40% of exposed applicators first used fonofos before 1980. Thus, although the period of cancer incidence follow-up is 7.5 years on average, the actual time from first use to the end of the follow-up period is longer. We did not observe an association between fonofos exposure and the incidence of all cancers combined. We did not have enough cases to evaluate NHL. There was, however, evidence of an association between fonofos and leukemia. There was also an observed association between fonofos and prostate cancer among those with a family history of prostate cancer.

Organophosphates have been associated with leukemia and other immunologically related cancers in the epidemiologic literature (Brown et al. 1990; Cantor et al. 1992; Clavel et al. 1996; De Roos et al. 2003; Lee et al. 2004; Waddell et al. 2001; Zahm et al. 1993). The leukemogenic effects of organophosphates may be related to immune function perturbation. Organophosphates irreversibly inhibit acetylcholine esterase, an enzyme that breaks down the neurotransmitter acetylcholine into inactive metabolites. Lymphocytes contain essential components of a cholinergic system, and studies suggest that prolonged acetylcholine esterase receptor stimulation, which could result from irreversible acetylcholine esterase inhibition, can alter lymphocytic activity (Kawashima and Fujii 2003).

Prostate cancer risk was not related to fonofos exposure overall. We did, however, find increased prostate cancer risk associated with fonofos use for those with a family history of prostate cancer. This result was previously reported in a case–control analysis of prostate cancer in the AHS, albeit with 87 fewer cases and 3.2 years shorter follow-up (Alavanja et al. 2003). Here we extend this result by also reporting a dose response with lifetime exposure-days and intensity-weighted exposure-days.

The statistical interaction that we observed here between fonofos exposure and family history of prostate cancer could have several explanations. One explanation may be that positive prostate cancer family history may serve as a surrogate for an inherited genetic trait, such as a polymorphism in a metabolic enzyme. There are known polymorphic variants of several cytochrome P450 isoforms that vary considerably in their ratio of chlorpyrifos bioactivation to detoxification (Dai et al. 2001; Tang et al. 2001). As organothiophosphates, fonofos and chlorpyrifos are similar in that they must be metabolized to their bio-active neurotoxic oxon forms (Maroni et al. 2000), and if fonofos shares some of the same metabolic enzymes as chlorpyrifos, such a polymorphism may account for the interaction. Alternatively, fonofos, phorate, and chlorpyrifos significantly inhibit testosterone metabolism in human liver microsomes, most likely as a result of their noncompetitive inhibition of cytochrome P450 3A4 testosterone metabolism (Usmani et al. 2003).

As with any study, some exposure misclassification is likely (Acquavella et al. 2006), but because exposure information was collected prospectively we have no reason to believe that it occurred differentially between cancer cases and cancer-free subjects. In addition, some of the exposure considered here may have occurred too recently to contribute to cancer occurrence. However, we repeated several analyses restricted to those whose year of first use occurred before 1990, and the results did not appreciably differ from the unrestricted analyses.

Pesticide applicators come into contact with multiple farm chemicals, including pesticides, and other agents. A previous AHS examination determined that a relationship between pesticide exposure and disease is not likely confounded by farming or nonfarming activities (Coble et al. 2002). In this study, we attempted to control for exposures to other pesticides using two approaches. First, we adjusted the risk estimates for use of the four pesticides that were most correlated with fonofos. Alternatively, to control for the effects of exposure to all pesticides, we adjusted for lifetime exposure-days to all pesticides. The measured differences between exposed and unexposed applicators in age and family history of cancer would normally raise concerns that these groups also differed with respect to other unmeasured cancer risk factors, but the overall conclusions did not differ when the unexposed and lowest exposed groups were used as the reference.

The main strengths of this study include its large prospective design, complete recruitment and follow-up, and the use of semi-quantitative exposure measures that improve on the qualitative measures used in previous studies of pesticide exposure. In addition, our results are internally consistent, as further adjustment and subgroup analyses did not result in different conclusions.

This is, to our knowledge, the largest examination of any group occupationally exposed to fonofos. Our strategy for evaluating the carcinogenic potential of pesticides in the cohort is to examine each pesticide with respect to cancer outcomes, to examine each cancer outcome with respect to pesticide exposures, and to examine the consistency of the relationship across time, state, and license type. Our conclusions are limited because of the small number of exposed cases, especially for leukemia. As follow-up of the cohort continues, more cancer cases will develop as the cohort ages, at which point the relationship between cancer and exposure to fonofos and other pesticides needs to be confirmed.

## Figures and Tables

**Table 1:** Characteristics of applicators by fonofos exposure in the AHS (1993–1997) [no. (%)].

Characteristic	Unexposed (*n* = 36,313)	Lowest exposed (*n* = 3,496)	Other exposed (*n* = 5,563)
Age (years)			
< 40	13,194 (36.3)	963 (27.6)	1,563 (28.1)
40–49	10,163 (28.0)	1,178 (33.7)	1,806 (32.5)
50–59	6,963 (19.2)	789 (22.6)	1,338 (24.1)
≥ 60	5,993 (16.5)	566 (16.2)	856 (15.4)
Sex			
Male	35,151 (96.8)	3,475 (99.4)	5,525 (99.3)
Female	1,162 (3.2)	21 (0.6)	38 (0.7)
State of residence			
Iowa	23,246 (64.0)	3,191 (91.3)	5,073 (91.2)
North Carolina	13,067 (36.0)	305 (8.7)	490 (8.8)
Applicator type			
Commercial	3,700 (10.2)	138 (4.0)	408 (7.3)
Private	32,613 (89.8)	3,358 (96.1)	5,155 (92.7)
Smoking history			
Never	19,957 (55.0)	2,033 (58.2)	3,232 (58.1)
Light ( ≤ 12 pack-years)	8,309 (22.9)	857 (24.5)	1,300 (23.4)
High ( ≥ 12 pack-years)	8,047 (22.2)	606 (17.3)	1,031 (18.5)
Education^a^			
≤ High school	19,270 (54.3)	1,858 (53.9)	3,078 (56.3)
> High school	16,214 (45.7)	1,591 (46.1)	2,387 (43.7)
Family history of cancer^a^			
No	21,353 (60.0)	1,919 (55.4)	3,064 (55.7)
Yes	14,228 (40.0)	1,545 (44.6)	2,442 (44.4)
Alcohol^a^^,^^b^			
No	11,666 (32.4)	781 (22.4)	1,185 (21.4)
Yes	24,373 (67.6)	2,700 (77.6)	4,353 (78.6)
Corn farming			
No	11,006 (30.3)	279 (8.0)	572 (10.3)
Yes	25,307 (69.7)	3,217 (92.0)	4,991 (89.7)
Use of correlated pesticides			
Trichorfon	264 (0.7)	26 (0.7)	99 (1.8)
Carbofuran	7,086 (19.5)	1,498 (42.9)	2,595 (46.6)
Imazethapyr	13,926 (38.4)	2,012 (57.6)	3,177 (57.1)
EPTC	6,055 (16.7)	1,091 (31.2)	1,962 (35.3)
Acres planted previous year^a^			
None	1,965 (6.1)	47 (1.4)	106 (2.0)
< 199	10,363 (32.1)	787 (23.2)	814 (15.4)
200–499	8,394 (26.0)	1,213 (35.8)	1,800 (34.0)
500–999	6,873 (21.3)	854 (25.2)	1,552 (29.3)
≥ 1,000	4,728 (14.6)	489 (14.4)	1,019 (19.3)
No. of livestock (other than poultry) on farm^a^			
None/did not farm	11,630 (36.3)	842 (24.9)	1,137 (21.5)
< 50	4,583 (14.3)	309 (9.2)	422 (8.0)
50–499	8,328 (26.0)	1,179 (34.9)	1,683 (31.9)
> 500	7,472 (23.3)	1,047 (31.0)	2,036 (38.6)
No. of pesticides used (mean ± SD)	11.7 ± 6.7	17.0 ± 6.7	18.4 ± 7.2

a Numbers of applicators do not sum to total because of missing information.

b Based on reported alcohol consumption in the previous 12 months.

**Table 2:** RRs^a^ (95% CIs) for selected cancers by fonofos intensity-weighted exposure-days among AHS (1993–1997) applicators, using unexposed and lowest-exposed applicators as the reference groups.

Intensity-weighted exposure-days	Cases (*n*)	Unexposed reference	Lowest exposed reference
All cancer			
0	1,514	1.00 (Referent)	
> 0–79	136	1.12 (0.93–1.35)	1.00 (Referent)
80–291	128	1.06 (0.88–1.28)	0.93 (0.73–1.19)
≥ 292	128	1.09 (0.90–1.32)	0.93 (0.72–1.21)
*p*_trend_		0.40	0.72
Prostate cancer			
0	588	1.00 (Referent)	
> 0–79	54	0.99 (0.74–1.32)	1.00 (Referent)
80–291	58	1.10 (0.83–1.46)	1.14 (0.78–1.65)
≥ 292	55	1.14 (0.86–1.53)	1.21 (0.81–1.79)
*p*_trend_		0.32	0.43
Lung cancer			
0	142	1.00 (Referent)	
> 0–79	10	1.54 (0.78–3.02)	1.00 (Referent)
80–291	5	0.64 (0.26–1.60)	0.40 (0.14–1.19)
≥ 292	8	0.93 (0.44–1.96)	0.66 (0.24–1.79)
*p*_trend_		0.70	0.75
Colon cancer			
0	112	1.00 (Referent)	
> 0–79	15	1.79 (1.01–3.18)	1.00 (Referent)
80–291	8	0.95 (0.45–2.00)	0.53 (0.22–1.26)
≥ 292	14	1.66 (0.92–3.03)	0.91 (0.42–2.01)
*p*_trend_		0.14	0.78
Lymphohematopoietic cancer			
0	151	1.00 (Referent)	
> 0–79	10	0.81 (0.42–1.56)	1.00 (Referent)
80–291	16	1.30 (0.76–2.22)	1.59 (0.72–3.52)
≥ 292	14	1.14 (0.64–2.02)	1.43 (0.61–3.36)
*p*_trend_		0.56	0.65
Leukemia			
0	47	1.00 (Referent)	
> 0–130.2	6	1.28 (0.52–3.10)	1.00 (Referent)
130.3–609	4	0.98 (0.34–2.82)	0.73 (0.20–2.62)
≥ 609	6	2.67 (1.06–6.70)	2.03 (0.58–7.05)
*p*_trend_		0.04	0.14
Melanoma skin cancer			
0	61	1.00 (Referent)	
> 0–79	8	1.52 (0.70–3.29)	1.00 (Referent)
80–291	3	0.60 (0.18–1.96)	0.40 (0.11–1.51)
≥ 292	6	1.17 (0.48–2.83)	0.88 (0.28–2.75)
*p*_trend_		0.86	0.86

a Adjusted for age (< 40, 40–49, 50–59, ≥ 60 years), state of residence, pack-years of smoking (0, ≤ 12, > 12), and use of the four most correlated pesticides (trichlorofon, carbofuran, imazethapyr, and EPTC).

**Table 3:** Prostate cancer RRs (95% CIs) among AHS (1993–1997) participants by family history of prostate cancer using fonofos lifetime and intensity-weighted exposure-days metrics.

	No family history		Family history		Interaction
Category	Cases (*n*)	RR^a^ (95% CI)	Cases (*n*)	RR^a^ (95% CI)	RR^b^ (95% CI)
Lifetime exposure-days^c^					
0	534	1.00 (Referent)	100	1.00 (Referent)	
> 0–20	58	1.08 (0.82–1.41)	16	1.42 (0.84–2.41)	
> 20–56	51	0.93 (0.70–1.25)	18	1.57 (0.95–2.60)	1.28 (1.07–1.54)
> 56	30	0.86 (0.60–1.24)	15	1.77 (1.03–3.05)	
*p*_trend_		0.37		0.02	
Intensity-weighted exposure-days					
0	534	1.00 (Referent)	100	1.00 (Referent)	
> 0–96	50	0.93 (0.69–1.24)	13	1.09 (0.61–1.95)	
> 97–314	45	1.03 (0.76–1.39)	17	1.94 (1.16–3.25)	1.27 (1.07–1.51)
> 315	42	0.96 (0.70–1.31)	19	1.83 (1.12–3.00)	
*p*_trend_		0.81		< 0.01	

a Adjusted for age (< 40, 40–49, 50–59, ≥ 60 years).

b Adjusted for age (< 40, 40–49, 50–59, ≥ 60 years) and family history of prostate cancer.

c For lifetime exposure-days, the categories generated based on the exposure distribution among prostate cancer cases happened to be the same as the categories generated from the exposure distribution of all cancer cases.
